# Miscellaneous Medical Intelligence

**Published:** 1848

**Authors:** 


### ARTICLE V.

### MISCELLANEOUS MEDICAL INTELLIGENCE.

Agreeably to notice given to the Commissioners of the Indiana Hospital for the Insane in October last, Dr. Jno. Evans resigned the office of Superintendent of that Institution, on the first day of July inst., and Dr. R. J. Patterson, for a number of years first assistant physician in the Ohio Lunatic Asylum, was appointed to the place. A portion of the institution will be opened for the reception of patients, about the first of August next.

The thanks of the Provisional Government of France have been tendered the physicians, surgeons, and apothecaries of the Hospitals, for their aid to the wounded during the revolution.

Accounts of the subsidence of the cholera in the East, have been circulated in most of our medical exchanges; but we observe by the newspapers, it is still progressing westward.

Prof. Bouilland has been appointed Dean of the faculty of Paris, in place of the venerable Ofila, removed, by the Provisional Government.

The Medical College of Georgia has extended the term of its course of lectures to five months. The term in this institution was six months for five years, from 1832 to 1837, which gives it the credit of priority in the move in reference to extending the term of lectures in Medical Schools.

Prof. Stromeyer has been appointed successor to Deiffenbach at Berlin.

Mr. Samuel Cooper, author of the Surgical Dictionary, and for 17 years Professor of Surgery in the University College, London, has resigned that chair on account of a difficulty with two of his colleagues.

